# Miss Ethel M. Warner and Mr. Lawson Tait

**Published:** 1892-12-24

**Authors:** Ethel M. Warner


### MISS ETHEL M. WARNER AND MR. LAWSON TAIT.

By Ethel M. Warner.

Mr. Lawson Tait, has again made a mistake; he will see, if he reads carefully through my letter in The Hospital of December 3rd, that it was not Mr. Godlee, but Dr. Macewen, who said, at the "meeting of the Royal Medical Chirurgical Society," that "the researches of Dr. Hughlings Jackson, Dr. Ferrier, and others had greatly assisted him" in planning and performing the operations which he described at the time that Mr. Godlee read a paper. In fact, he thus acknowledged his indebtedness to the experiments, which Mr. Tait thinks had no place in his conclusions at all. It is a noteworthy fact, that the "anti-vivisectionists, after stating what is untrue, object and feel hurt when they are told that their statements are "false"; and I note that Mr. Lawson Tait does not differ in this respect from the rest of the "society" to which he belongs.

